# Patterns of diuretic use in the intensive care unit

**DOI:** 10.1371/journal.pone.0217911

**Published:** 2019-05-31

**Authors:** Ian Ellis McCoy, Glenn Matthew Chertow, Tara I-Hsin Chang

**Affiliations:** Division of Nephrology, Stanford University School of Medicine, Palo Alto, California; University of Sao Paulo Medical School, BRAZIL

## Abstract

**Purpose:**

To inform future outcomes research on diuretics, we sought to describe modern patterns of diuretic use in the intensive care unit (ICU), including diuretic type, combination, and dosing. We also investigated two possible quality improvement targets: furosemide dosing in renal impairment and inclusion of an initial bolus with continuous furosemide infusions.

**Materials and methods:**

In this descriptive study, we retrospectively studied 46,037 adult ICU admissions from a publicly available database of patients in an urban, academic medical center.

**Results:**

Diuretics were employed in nearly half (49%, 22,569/46,037) of ICU admissions. Mechanical ventilation, a history of heart failure, and admission to the post-cardiac surgery unit were associated with a higher frequency of diuretic use. Combination use of different diuretic classes was uncommon. Patients with severely impaired kidney function were less likely to receive diuretics. Furosemide was by far the most common diuretic given and the initial intravenous dose was only 20 mg in more than half of ICU admissions. Among patients treated with a continuous infusion, 30% did not receive a bolus on the day of infusion initiation.

**Conclusions:**

Patterns of diuretic use varied by patient-specific factors and by ICU type. Diuretic dosing strategies may be suboptimal.

## Introduction

Fluid management is one of the most challenging clinical problems in the intensive care unit (ICU). While some patients present with fluid overload, other patients acquire fluid overload after admission to the ICU due to administration of intravenous fluid therapy, which is often the initial treatment maneuver for hypotension of any cause [1,2]. Diuretics are a mainstay for managing fluid overload and are commonly prescribed in the ICUs of all types [3–5]. However, there are few guidelines regarding the selection and combination of different diuretic classes, the choice of initial dosages, or the timing of initiation during a patient’s clinical course [6]. Providers from different specialties may have significant variation in diuretic practice patterns.

Despite the widespread use of diuretics, few studies have examined patterns of diuretic use in the ICU. A 2004 prevalence study of ICUs in France found that 49% of ICU patients received diuretics, mostly intravenous furosemide [3]. A retrospective study of 10 ICUs at sites across the United Kingdom and Canada showed wide variability (15–45%) in furosemide use [5].

Understanding current diuretic practice patterns will be critical in the design of future outcome trials aimed at optimizing diuretic strategies in critical illness. Furthermore, an investigation of modern diuretic dosing strategies may identify opportunities for quality improvement. For example, continuous diuretic infusions begun without a bolus will take many hours to reach an effective serum concentration, and higher diuretic doses are required in patients with decreased renal function to achieve an equivalent diuretic effect. It is unknown whether and to what extent current diuretic prescribing practices consider these pharmacologic principles.

We sought to describe patterns of diuretic use, including diuretic type and combination, dosing and route of administration, among different clinical settings using contemporary data from a large urban U.S. academic medical center. We analyzed ICU diuretic practice patterns during a 10-year period and report associations with patient-specific factors and ICU type.

## Materials and methods

### Data source (MIMIC-III)

We analyzed de-identified data from the publicly available Medical Information Mart for Intensive Care (MIMIC-III) database v1.4. MIMIC-III is managed by the Massachusetts Institute of Technology (MIT) Laboratory for Computational Physiology and contains data on over 40,000 ICU patients at the Beth Israel Deaconess Medical Center (BIDMC) between 2001 and 2012 [7,8]. Beth Israel Deaconess Medical Center is a 700-bed, 77-adult ICU bed, academic medical center in Boston, MA affiliated with Harvard Medical School. The database was approved for research by the Institutional Review Boards of MIT and BIDMC and studies using the database are granted a waiver of informed consent due to its de-identified nature.

From the total 53,366 ICU admissions where the patient was at least 18 years old at the time of hospital admission, we selected the 48,679 (91%) ICU admissions where medication order information was available. We excluded 2,642 ICU admissions with an indicator of end-stage renal disease (ESRD), defined as having an International Classification of Diseases, Ninth Revision (ICD-9) diagnosis code for ESRD (585.6) or having an ICD-9 code for a dialysis procedure (39.95 for hemodialysis or 54.98 for peritoneal dialysis) without an ICD-9 code for acute kidney injury (AKI) (584.5, 584.6, 584.7, 584.8, 584.9). Our final study cohort contained 46,037 ICU admissions. We conducted an additional sensitivity analysis where we removed all ICU admissions with any dialysis procedure (N = 958, 2%).

A new ICU admission was created if the patient was out of the ICU for more than 24 hours before returning. We included multiple ICU admissions from the same patient and the same hospitalization (46,037 ICU admissions, 42,981 total hospitalizations, 34,331 total patients). Since the age of patients over age 89 was removed previously during the de-identification process when the database was created, we set the age of these patients to 90. We changed variable values that were implausible to missing (8 admission weight > 500 kg, 20 admission serum creatinine concentrations > 20 mg/dL, 296 length of stay (LOS) fluid balances over 80 L net positive or negative).

### Key dependent and independent variables

We defined admission serum creatinine concentration as the first serum creatinine measured during the hospital admission. We captured comorbidities from ICD-9 codes billed for each hospital admission according to version 3.7 of the Elixhauser comorbidities defined by the Agency for Healthcare Research and Quality (AHRQ) [9]. We categorized admission type based on the primary ICD-9 code for each admission as due to cardiovascular, gastrointestinal, infectious, respiratory, or malignant disease, injury/poisoning, or other causes [10]. We ascertained each of the five ICU types: Medical, Surgical, Post-Cardiac Surgical, Cardiac, and Trauma. ICU type refers to the physical ICU where the patient was admitted. The medical and cardiac ICUs, are typically staffed by medical intensivists and cardiologists, respectively, while the surgical, post-cardiac surgical, and trauma ICUs are typically staffed by surgeons or anesthesiologists. We calculated fluid balance as all inputs minus all outputs during the ICU stay.

We categorized diuretics as follows: loop diuretics (bumetanide, ethycrinic acid/ethycrinate sodium, furosemide, torsemide), thiazides/thiazide-type diuretics (chlorthalidone, chlorothiazide, hydrochlorothiazide, indapamide, metolazone), mineralocorticoid receptor antagonists (MRAs) (eplerenone, spironolactone), carbonic anhydrase inhibitors (CAIs) (acetazolamide), and epithelial sodium channel blockers (amiloride, triamterene). We considered combination diuretic use as prescription of two or more diuretic classes during the ICU stay, but not necessarily on the same day (i.e. prescription of a loop diuretic on day 1 followed by prescription of a thiazide diuretic on day 2 would have been counted as a loop + thiazide combination). We did not consider the use of two diuretics of the same class (e.g., furosemide and bumetanide) as combination diuretic use.

### Statistical analysis

We compared continuous variables using analysis of variance (ANOVA) and categorical variables using the χ^2^ test. To assess the association of candidate variables with the use (versus non-use) of: any diuretic, a specific diuretic class or a specific combination of diuretics in separate models, we used logistic regression. Adjusted models include age, sex, race, comorbidities (hypertension, heart failure, chronic kidney disease, diabetes, liver disease), admission type, ICU type, mechanical ventilation, and admission serum creatinine concentration category. After noting a difference in carbonic anhydrase inhibitor use by ICU type, we conducted a separate sensitivity analysis, where we included maximum serum bicarbonate levels on ICU day 1 in the models. We report odds ratios (OR) and 95% confidence intervals (CI). We used the concordance “c” statistic as an assessment of model discrimination and the Hosmer-Lemeshow goodness-of-fit test to evaluate for model calibration. Given the very small proportion of missing data (<0.3% of observations had missing data for the variables in the models), we conducted a complete case analysis.

We used Spearman correlation coefficients to estimate associations among patient body size and serum creatinine with furosemide dosing. We considered 2-sided p-values < 0.05 as statistically significant. We performed all statistical analyses using SAS software, version 9.4 (SAS Institute, Cary, NC).

## Results

We identified 46,037 adult ICU stays. **Table 1** shows patient characteristics for these ICU stays within each of the five ICU types (Medical, Surgical, Post-Cardiac Surgical, Cardiac, and Trauma). The mean age was 64.2 years, 43.6% were female, 2.4%, 8.5%, and 3.4% were Asian, Black, and Hispanic respectively. More than one in eight (12.7%) patients had a history of chronic kidney disease (CKD), more than half (54.6%) had a history of hypertension, and more than a quarter (27.8%) had a history of heart failure. The mean (± SD) serum creatinine concentration on hospital admission was 1.3 ± 1.0 mg/dL. Cardiovascular and injury/poisoning diagnoses were the most common reasons for admission overall (35.1% and 16.4% respectively).

**Table 1:** Characteristics of ICU stays in each ICU type*†‡.

	Medical (N = 18128)	Surgical (N = 7757)	Post-Cardiac Surgical (N = 7740)	Cardiac (N = 6451)	Trauma (N = 5961)
**Age (years)**	63.9 +/- 17.8	63.2 +/- 16.6	67.0 +/- 12.9	68.5 +/- 15.3	58.2 +/- 20.7
**Female sex**	48.2%	46.6%	34.3%	42.2%	39.1%
**Race/Ethnicity**					
Asian	2.9%	2.7%	1.8%	1.8%	2.0%
Black	12.4%	7.9%	3.4%	6.9%	5.6%
Hispanic	3.8%	3.6%	2.7%	2.3%	4.1%
Other	2.5%	3.3%	2.5%	2.3%	3.6%
Unknown	6.3%	6.0%	14.4%	12.8%	9.9%
White	72.1%	76.5%	75.2%	73.9%	74.8%
**Comorbidities**					
Diabetes mellitus	28.6%	23.9%	30.0%	32.4%	17.4%
Heart failure	29.6%	15.8%	28.3%	51.0%	11.8%
Hypertension	50.8%	53.8%	68.6%	61.6%	41.4%
Chronic kidney disease	15.6%	9.1%	9.5%	19.0%	5.7%
Liver disease	12.6%	12.0%	2.6%	4.4%	4.5%
Metastatic cancer	8.7%	9.4%	1.3%	3.0%	5.8%
**Admit type**					
Cardiovascular	12.2%	27.6%	84.5%	71.0%	12.0%
Gastrointestinal	16.1%	16.0%	1.2%	3.4%	8.9%
Infectious	16.9%	5.8%	0.8%	4.5%	3.3%
Respiratory	17.5%	5.3%	1.7%	5.8%	4.2%
Neoplasm	7.9%	16.3%	2.8%	2.0%	9.0%
Injuries/Poisonings	11.2%	17.4%	5.7%	7.1%	55.0%
Other	18.2%	11.6%	3.3%	6.2%	7.6%
**Admission serum creatinine (mg/dL)**	1.4 +/- 1.3	1.1 +/- 0.9	1.0 +/- 0.6	1.4 +/- 1.0	1.1 +/- 0.7
**Admission weight (kg)**	80.2 +/- 25.2	79.8 +/- 23.6	82.9 +/- 20.0	82.1 +/- 24.1	80.8 +/- 23.5
**First day urine output (L)**	1.9 +/- 1.4	1.9 +/- 1.2	2.2 +/- 1.1	2.0 +/- 1.4	2.0 +/- 1.3
**First day fluid balance (L)**	2.4 +/- 3.7	2.6 +/- 4.3	3.7 +/- 3.6	0.8 +/- 2.7	3.3 +/- 4.4
**ICU LOS (days)**	4.0 +/- 5.7	4.7 +/- 6.8	3.7 +/- 5.7	3.9 +/- 5.6	4.5 +/- 6.6
**ICU LOS fluid balance (L)**	4.5 +/- 8.9	5.6 +/- 9.9	3.1 +/- 6.4	1.6+/-7.6	6.4 +/- 9.7
**Mechanically ventilated**	37.0%	47.7%	86.7%	31.9%	54.3%

*Categorical variables are given as percentages of each ICU type. Continuous variables are expressed as mean +/- standard deviation.

†Missingness was < 0.4% for admission serum creatinine and 3–6% for fluid balance variables for all ICU types. For admission weight, missingness was 7.3%, 11.2%, 2.4%, 9.7%, and 12.4% in the medical, surgical, post-cardiac surgical, cardiac, and trauma units respectively.

‡ All differences between the five ICUs were statistically significant by ANOVA or chi-squared testing.

Patients admitted to the medical and cardiac ICUs had higher admission serum creatinine concentrations and were more likely to have a history of CKD compared to patients admitted to surgical, post-cardiac surgical, and trauma ICUs. Patients admitted to medical and cardiac ICUs also had lower rates of mechanical ventilation.

Nearly half (49.0%) of the ICU admissions had evidence of diuretic use. The most common diuretic used was furosemide (94.4% of ICU stays with diuretic administration). Combination diuretic strategies were uncommon as 77.9% of ICU admissions with any diuretic use received a loop diuretic alone (**S1 Fig**).

### Factors associated with diuretic use

**Fig 1:**
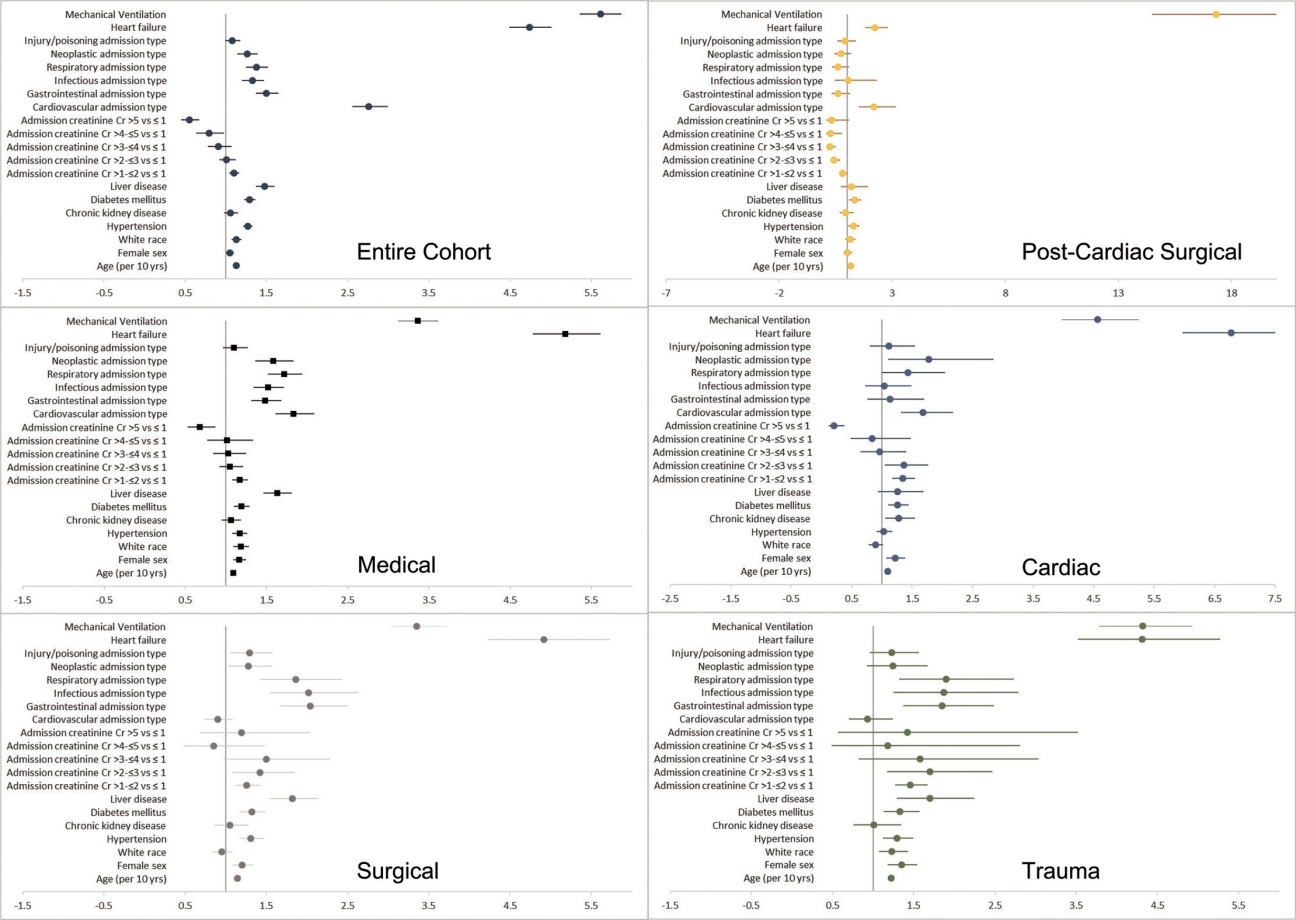
Forest plot of adjusted odds ratios for diuretic use, stratified by ICU type. The reference groups for admission type and admission serum creatinine category were ‘Other’ category admission type and admission serum creatinine ≤ 1 mg/dL, respectively. Odds ratios were calculated from a model containing age, sex, race, comorbidities (hypertension, heart failure, CKD, diabetes and liver disease), admission type, mechanical ventilation and admission serum creatinine category.

**Fig 2:**
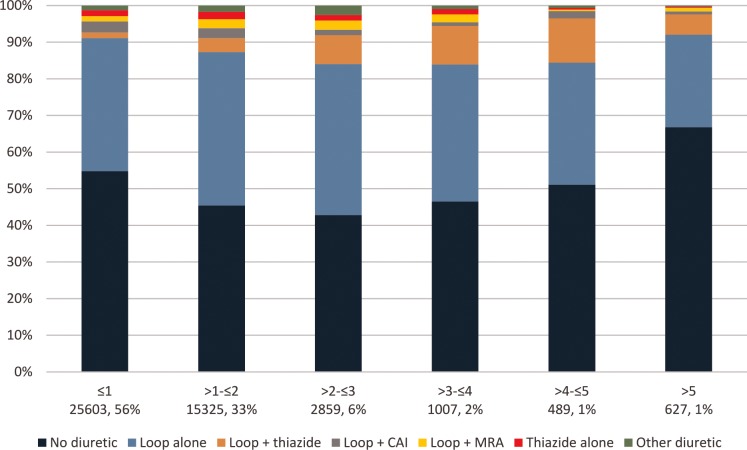
Diuretic use by admission serum creatinine. Column labels show the admission serum creatinine in mg/dL, listed above the number of ICU admissions in the group as N. There were 127 ICU admissions without a serum creatinine measurement.

Admission to the post-cardiac surgical ICU (versus medical ICU), mechanical ventilation, and a history of heart failure were among the factors associated with diuretic use (adjusted ORs 8.49, 4.21, and 5.09, respectively; **S1 Table**). We also saw higher adjusted odds of diuretic use in patients with a history of hypertension, diabetes, or liver disease. Relative to other admission diagnoses, patients with cardiovascular, respiratory, infectious, neoplastic, or injury/poisoning-related hospitalizations were more likely to receive diuretics during the ICU stay. Associations between patient factors and diuretic use varied widely by ICU type (**Fig 1**). For example, the association between mechanical ventilation and diuretic use was 5-fold larger in the post-cardiac surgical ICU than it was in the medical ICU. Diuretic use also varied with admission serum creatinine. Diuretic use increased with higher admission serum creatinine up to a serum creatinine concentration of 3 mg/dL, after which diuretic use decreased (**Fig 2**). This non-monotonic trend was materially unchanged when we removed patients from the cohort who received renal replacement therapy (2%) (data not shown), with a similar fraction of patients with serum creatinine > 5 mg/dL never having received diuretics (70% vs 67%).

### ICU type and diuretic use

**Fig 3:**
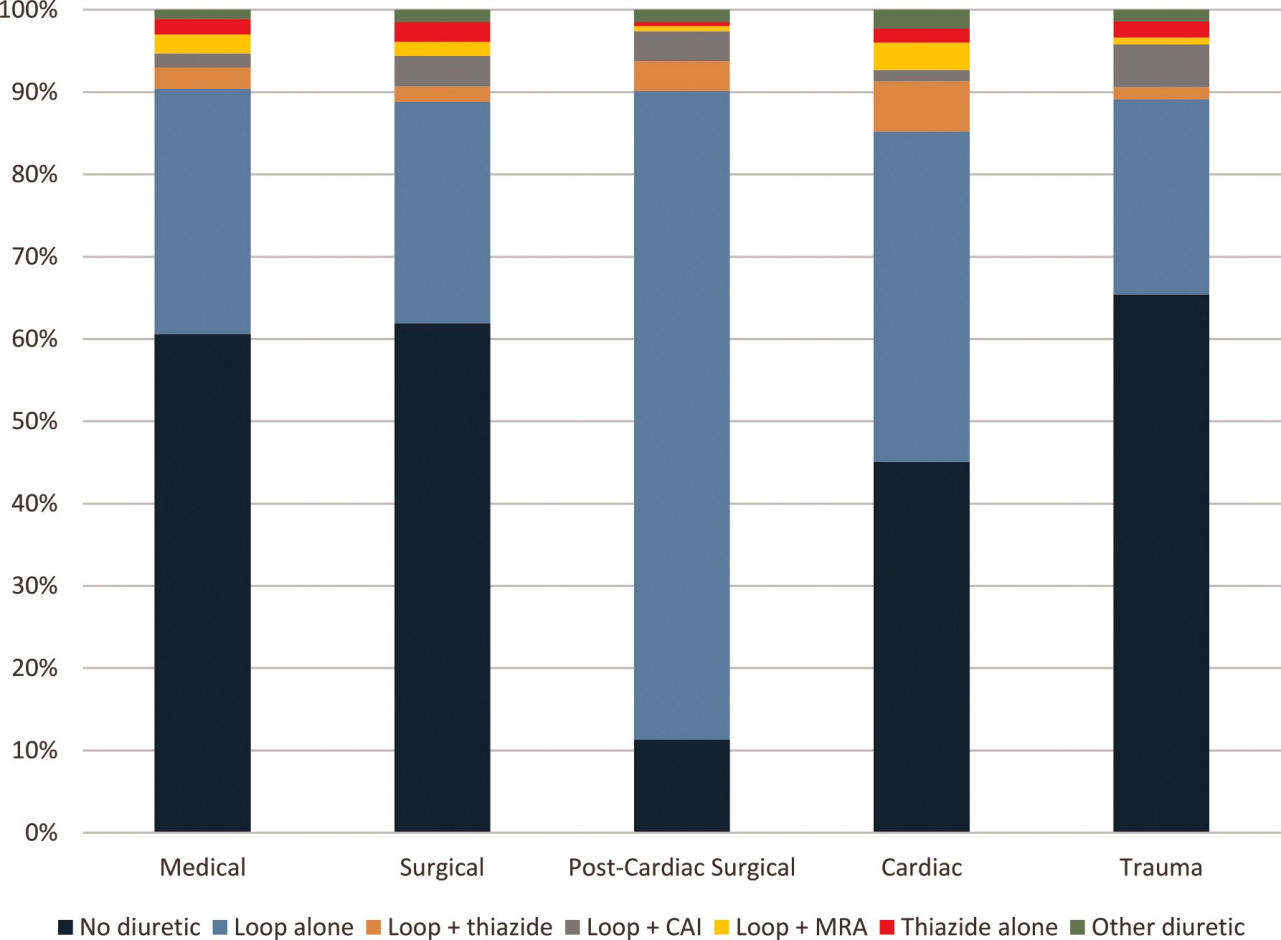
Diuretic use by ICU type.

Diuretic use and diuretic combination use differed depending on the ICU type (**Fig 3**). Post-cardiac surgical unit admissions were more likely to receive diuretics, and diuretic use in this unit consisted mostly of loop diuretics alone (89%). Surgical ICU (surgical, post-cardiac surgical, and trauma) admissions were more likely to include carbonic anhydrase inhibitors than were medical ICU (medical and cardiac) admissions (adjusted OR 1.89, 95% CI 1.68–2.14; **S2 Table**). In fact, carbonic anhydrase inhibitor use was highest in the trauma ICU, where the odds of receiving a carbonic anhydrase inhibitor were twice what they were in the medical ICU (adjusted OR 2.65, 95% CI 2.24–3.13). There were no significant differences in serum bicarbonate levels between surgical and medical ICUs (median 25 mEq/L in both ICU types, p = 0.57). Cardiac ICU admissions had the highest odds of receiving the loop + thiazide diuretic combination (adjusted OR 1.79, 95% CI 1.52–2.11, with the medical ICU as the reference group; **S3 Table**).

### Initial IV furosemide dosing

An analysis of the first IV furosemide bolus dose given during each ICU admission showed statistically significant, but weak, direct correlations between admission serum creatinine and dose (r = 0.26, p<0.0001) and between body weight and dose (r = 0.12, p<0.0001). The range of IV furosemide doses administered was narrow with 91% of patients receiving one of three doses: 58% received 20 mg, 17% received 40 mg, and 16% received 10 mg. The maximum initial dose in the cohort was 200 mg, with a median dose of 20 mg, a mean dose of 27 mg, and a standard deviation of 23 mg. When stratified into the admission serum creatinine categories, the median dose was 20 mg for ICU admissions with admission serum creatinines ≤ 1, >1-≤2, and >2-≤3 mg/dL and 40 mg for ICU admissions with admission serum creatinines >3-≤4, >4-≤5, and > 5 mg/dL. In fact, 5% of those with an admission serum creatinine > 5 mg/dL were still initially dosed with only 10 mg of intravenous furosemide.

Continuous intravenous furosemide infusions were used in 14% of ICU admissions with any intravenous furosemide use. Among those treated with continuous intravenous furosemide infusions, 30% did not receive a bolus on the day of infusion initiation.

### Specific diuretics and patient-specific factors

Intensive care unit admissions with mechanical ventilation had higher odds of carbonic anhydrase inhibitor use (adjusted OR 14.10, 95% CI 11.66–17.06; **S2 Table**). This association was materially unchanged when serum bicarbonate was added to the model for patients in whom the data were available (44,794/46,037 had bicarbonate data available; adjusted OR 14.48, 95% CI 11.97–17.52). The odds of loop + thiazide combination diuretic use were higher among patients with higher admission serum creatinine (adjusted OR 6.09, 95% CI 4.41–8.41, for admission serum creatinine concentration >4-≤5 versus ≤1 mg/dL; **S3 Table**). Patients with a history of heart failure also had higher odds of receiving loop + thiazide combination diuretics (adjusted OR 2.60, 95% CI 2.29–2.95). A history of liver disease was the factor most strongly associated with mineralocorticoid antagonist use (adjusted OR 10.25, 95% CI 8.87–11.85).

## Discussion

Using a large, contemporary database, we found that patterns of diuretic use varied by patient characteristics and by ICU type. Admission to the post-cardiac surgery ICU remained the strongest predictor of diuretic use, even after adjustment for clinical factors often associated with fluid overload, including mechanical ventilation and heart failure. Mechanical ventilation was strongly associated with diuretic use, especially carbonic anhydrase inhibitor use. The increased use of carbonic anhydrase inhibitors with mechanical ventilation may be related to concerns of adverse effects of metabolic alkalosis in this population [11], despite recent evidence that carbonic anhydrase inhibitors may not have clinical benefits such as decreased duration of mechanical ventilation [12,13]. The greater than 2-fold higher prevalence of carbonic anhydrase inhibitor usage in surgical ICUs as compared with medical ICUs cannot be entirely explained by the higher rate of mechanical ventilation in surgical ICUs and may reflect different paradigms for responding to metabolic alkalosis among specialties [14].

We also found that when the admission serum creatinine concentration was ≤ 3 mg/dL, diuretic use directly correlated with serum creatinine concentration, which may reflect the fact that patients with impaired kidney function are more likely to have fluid overload. However, with admission serum creatinine concentrations > 3 mg/dL, an increasing proportion of patients never received diuretics (67% for those with an admission serum creatinine concentration > 5 mg/dL). Removal of patients treated with renal replacement therapy did not alter this trend. Although we cannot ascertain the clinical reasons for diuretic ordering decisions, we postulate that our findings may reflect provider perceptions that diuretics may be futile in patients with severely impaired kidney function or that diuretics may impair kidney function further.

Combination diuretic use was rare, though most common in the cardiac unit where the loop + thiazide combination was given in 6.1% of ICU admissions. This finding may reflect concern over possible adverse effects of this strategy including AKI, hyponatremia, and hypokalemia, despite recommendations for “sequential nephron blockade” as a means of overcoming loop diuretic resistance [15–17]. Indeed, the risks and benefits of adding thiazide diuretics in loop diuretic resistance remain largely unknown.

Patients with lower glomerular filtration rates are known to need higher doses of furosemide to achieve the same diuretic effect [15,18,19]. Patients with higher serum creatinine concentrations may need IV furosemide doses of 200 mg or more to be maximally effective [20]. Although initial IV furosemide bolus dose was statistically correlated with admission creatinine in our study, the absolute increase in dose for patients with higher creatinine was small–the median dose ordered with creatinines > 5 mg/dL was only 20 mg higher than the median dose ordered with creatinines ≤ 1 mg/dL (40 mg versus 20 mg). A multinational survey of intensivists and nephrologists found that, in determining diuretic dose, serum creatinine was rated as ‘important’ or ‘very important’ by 74% [21]. However, our results suggest that, in determining initial diuretic doses, consideration of kidney function is likely limited.

An initial bolus or ‘loading dose’ allows continuous loop diuretic infusions to work rapidly as soon as a clinician makes the decision to diurese [22–24]. Continuous diuretic infusions begun without a bolus will take many hours to reach an effective serum diuretic concentration, yet in our study 30% of infusions were begun without a bolus. These findings suggest that diuretic prescribing practices in the ICU may be suboptimal and should be investigated in more detail and in other datasets.

Our study has several strengths. The cohort is large with more than 40,000 ICU admissions across five different ICU types in an urban medical center. Furthermore, the data are extremely detailed, abstracted directly from the ICU electronic health record, with detailed quantitative data on drug prescription and kidney function. Limitations of our study include the single-center data source and reliance on administrative ICD-9 codes for comorbidities and reasons for admission. The ICU admissions in the cohort were not completely independent given the inclusion of multiple ICU admissions from the same hospitalization and the same patient. Home medications for each ICU admission were not available. Finally, we were not able to ascertain clinical reasons underlying decisions about whether to use diuretics, what type of diuretic to use, and at what dose.

In summary, our study provides a contemporary description of diuretic use and combination diuretic use in critically ill patients, which may inform the design of future studies evaluating the relative safety and effectiveness of various diuretic strategies. For example, our finding of marked variation in diuretic use between ICU types establishes ICU type as a key potential confounder in clinical studies of diuretic use. Also, power calculations and recruitment planning for future experimental trials of diuretics in the ICU may be aided by the prevalences of different patterns of diuretic use presented here. We identified significant variation in diuretic use by patient characteristics and by ICU type. We also found probable under-dosing of diuretics in patients with CKD (in terms of dose) and among patients receiving loop diuretic infusions with regard to bolus prescription, which are potential targets for quality improvement efforts. Our results suggest that diuretic dosing strategies in the ICU may be suboptimal, and future studies are needed to identify ways to improve their use.

## Supporting information

S1 Fig Distribution of diuretic combinations among ICU stays with at least one diuretic. MRA: mineralocorticoid receptor antagonist; CAI: carbonic anhydrase inhibitor.
(TIF)

S1 Table Odds ratios for diuretic use. The reference groups for ICU type, admission type, and admission serum creatinine were medical unit, ‘Other’ category admission type, and admission serum creatinine ≤ 1 mg/dL, respectively. Adjusted odds ratios were calculated from a model including age, sex, race, ICU type, admission type, mechanical ventilation, comorbidities (hypertension, heart failure, CKD, diabetes and liver disease), and admission creatinine category.
(DOCX)

S2 Table Odds ratios for carbonic anhydrase use. The reference groups for ICU type, admission type, and admission serum creatinine were medical unit, ‘Other’ category admission type, and admission serum creatinine ≤ 1 mg/dL, respectively. Surgical ICUs are post-cardiac surgical, surgical, and trauma; medical ICUs refer to medical and cardiac. Adjusted odds ratios were calculated from a model including age, sex, race, ICU type, admission type, mechanical ventilation, comorbidities (hypertension, heart failure, CKD, diabetes and liver disease), and admission creatinine category.
(DOCX)

S3 Table Odds ratios for loop + thiazide use. The reference groups for ICU type, admission type, and admission serum creatinine were medical unit, ‘Other’ category admission type, and admission serum creatinine ≤ 1 mg/dL, respectively. Adjusted odds ratios were calculated from a model including age, sex, race, ICU type, admission type, mechanical ventilation, comorbidities (hypertension, heart failure, CKD, diabetes and liver disease), and admission creatinine category.
(DOCX)
