# Supplementary figures and images for: Patterns of diuretic use in the intensive care unit
Source: PLoS One. 2019 May 31;14(5):e0217911. doi: 10.1371/journal.pone.0217911 (PMC6544280; doi:10.1371/journal.pone.0217911)

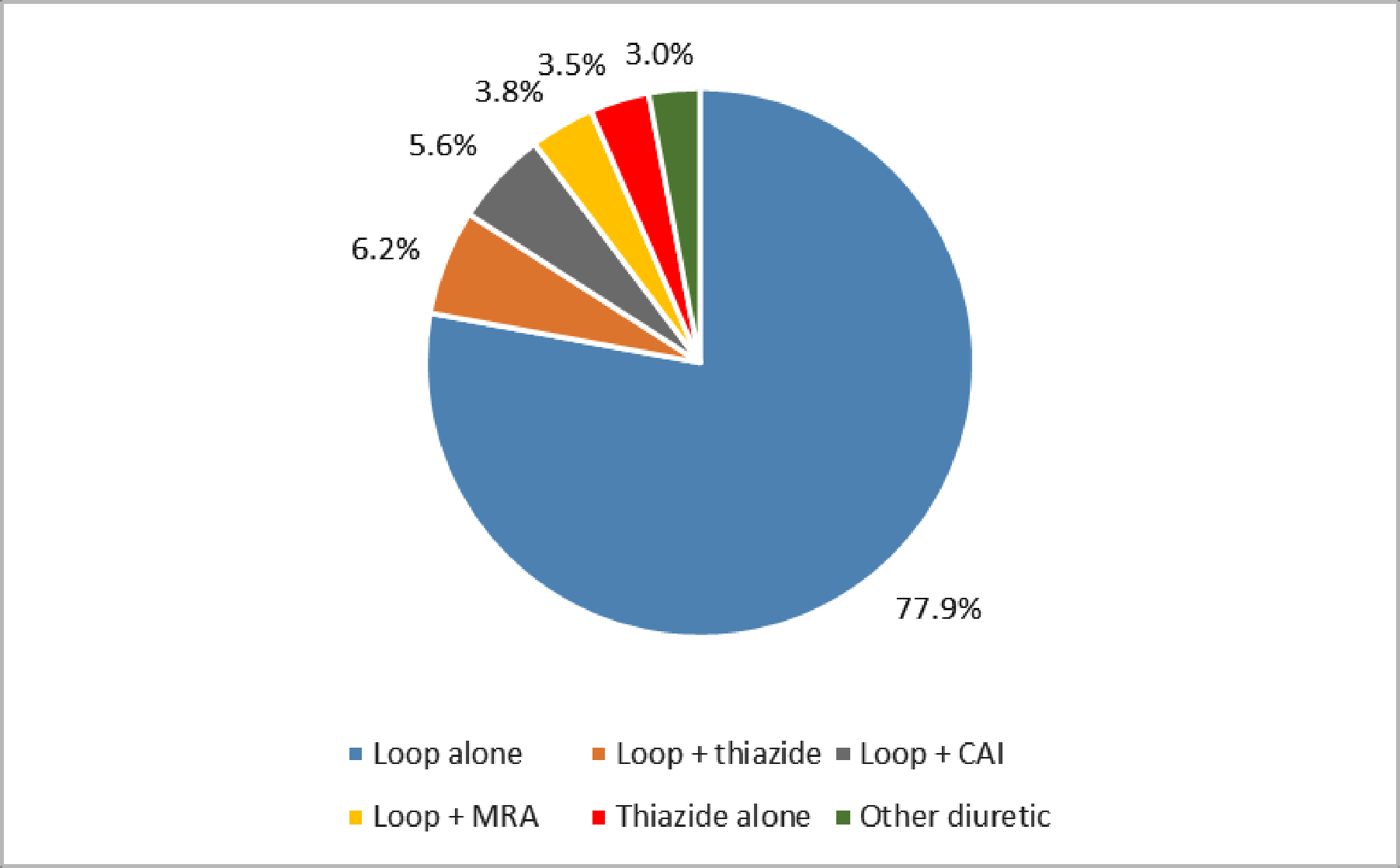

Supplement: S1 Fig — MRA: mineralocorticoid receptor antagonist; CAI: carbonic anhydrase inhibitor. (TIF) [file pone.0217911.s001.tif]
