# Supplementary material for: Patterns of diuretic use in the intensive care unit
Source: PLoS One. 2019 May 31;14(5):e0217911. doi: 10.1371/journal.pone.0217911 (PMC6544280; doi:10.1371/journal.pone.0217911)
Supplement: S1 Table — The reference groups for ICU type, admission type, and admission serum creatinine were medical unit, ‘Other’ category admission type, and admission serum creatinine ≤ 1 mg/dL, respectively. Adjusted odds ratios were calculated from a model including age, sex, race, ICU type, admission type, mechanical ventilation, comorbidities (hypertension, heart failure, CKD, diabetes and liver disease), and admission creatinine category. (DOCX) [file pone.0217911.s002.docx]

**S1 Table.** Odds ratios for diuretic use

| **Column1** | **OR for Diuretic Use** | **95% CI** | **Adjusted OR** | **95% CI** |
| --- | --- | --- | --- | --- |
| **Post-Cardiac Surgical Unit admission** | 12.08 | 11.19-13.04 | 8.49 | 7.73-9.33 |
| **Heart failure** | 5.03 | 4.81-5.27 | 5.09 | 4.80-5.38 |
| **Mechanical Ventilation** | 4.60 | 4.42-4.78 | 4.21 | 4.02-4.42 |
| **Cardiovascular admission type** | 4.48 | 4.19-4.79 | 1.56 | 1.43-1.70 |
| **Respiratory admission type** | 2.58 | 2.38-2.81 | 1.57 | 1.43-1.74 |
| **Infectious admission type** | 2.03 | 1.86-2.21 | 1.48 | 1.34-1.63 |
| **Cardiac Unit admission** | 1.87 | 1.77-1.98 | 1.48 | 1.38-1.60 |
| **Hypertension** | 1.77 | 1.71-1.84 | 1.20 | 1.14-1.26 |
| **Chronic kidney disease** | 1.69 | 1.60-1.79 | 1.06 | 0.98-1.15 |
| **Gastrointestinal admission type** | 1.68 | 1.55-1.82 | 1.63 | 1.48-1.79 |
| **Admission creatinine Cr >2-≤3 vs ≤ 1** | 1.62 | 1.50-1.75 | 1.16 | 1.05-1.28 |
| **Diabetes mellitus** | 1.61 | 1.54-1.68 | 1.27 | 1.20-1.34 |
| **Admission creatinine Cr >1-≤2 vs ≤ 1** | 1.46 | 1.40-1.52 | 1.20 | 1.14-1.27 |
| **Admission creatinine Cr >3-≤4 vs ≤ 1** | 1.40 | 1.23-1.59 | 1.05 | 0.90-1.23 |
| **Neoplastic admission type** | 1.32 | 1.21-1.45 | 1.33 | 1.20-1.48 |
| **Age (per 10 years)** | 1.30 | 1.29-1.32 | 1.13 | 1.11-1.15 |
| **Injury/Poisoning admission type** | 1.21 | 1.13-1.31 | 1.14 | 1.04-1.25 |
| **Admission creatinine Cr >4-≤5 vs ≤ 1** | 1.16 | 0.97-1.39 | 0.91 | 0.74-1.13 |
| **White race** | 1.13 | 1.08-1.17 | 1.10 | 1.05-1.21 |
| **Female sex** | 0.99 | 0.95-1.02 | 1.16 | 1.11-1.21 |
| **Surgical Unit admission** | 0.95 | 0.90-1.00 | 1.05 | 0.98-1.12 |
| **Liver disease** | 0.90 | 0.84-0.96 | 1.65 | 1.52-1.78 |
| **Trauma Unit admission** | 0.82 | 0.77-0.87 | 1.09 | 1.01-1.18 |
| **Admission creatinine Cr >5 vs ≤ 1** | 0.60 | 0.51-0.71 | 0.63 | 0.51-0.77 |

The reference groups for ICU type, admission type, and admission serum creatinine were medical unit, ‘Other’ category admission type, and admission serum creatinine ≤ 1 mg/dL, respectively. Adjusted odds ratios were calculated from a model including age, sex, race, ICU type, admission type, mechanical ventilation, comorbidities (hypertension, heart failure, CKD, diabetes and liver disease), and admission creatinine category.
