# Supplementary material for: Patterns of diuretic use in the intensive care unit
Source: PLoS One. 2019 May 31;14(5):e0217911. doi: 10.1371/journal.pone.0217911 (PMC6544280; doi:10.1371/journal.pone.0217911)
Supplement: S2 Table — The reference groups for ICU type, admission type, and admission serum creatinine were medical unit, ‘Other’ category admission type, and admission serum creatinine ≤ 1 mg/dL, respectively. Surgical ICUs are post-cardiac surgical, surgical, and trauma; medical ICUs refer to medical and cardiac. Adjusted odds ratios were calculated from a model including age, sex, race, ICU type, admission type, mechanical ventilation, comorbidities (hypertension, heart failure, CKD, diabetes and liver disease), and admission creatinine category. (DOCX) [file pone.0217911.s003.docx]

**S2 Table**. Odds ratios for carbonic anhydrase use

| **Column1** | **OR for CAI Use** | **95% CI** | **Adjusted OR** | **95% CI** |
| --- | --- | --- | --- | --- |
| **Mechanical Ventilation** | 14.76 | 12.24-17.78 | 14.10 | 11.66-17.06 |
| **Respiratory admission type** | 3.25 | 2.52-4.18 | 1.92 | 1.48-2.50 |
| **Injury/Poisoning admission type** | 3.13 | 2.47-3.97 | 1.87 | 1.46-2.40 |
| **Trauma Unit admission** | 2.89 | 2.50-3.33 | 2.65 | 2.24-3.13 |
| **Gastrointestinal admission type** | 2.57 | 1.99-3.31 | 2.31 | 1.77-3.01 |
| **Infectious admission type** | 2.38 | 1.82-3.12 | 1.99 | 1.50-2.63 |
| **Surgical vs Medical ICUs** | 2.34 | 2.11-2.59 | 1.89 | 1.68-2.14 |
| **Cardiovascular admission type** | 2.22 | 1.76-2.79 | 1.18 | 0.92-1.52 |
| **Post-Cardiac Surgical Unit admission** | 2.18 | 1.89-2.52 | 1.40 | 1.16-1.69 |
| **Surgical Unit admission** | 2.17 | 1.88-2.51 | 2.21 | 1.89-2.59 |
| **Heart failure** | 1.92 | 1.74-2.12 | 2.19 | 1.95-2.46 |
| **Neoplastic admission type** | 1.76 | 1.32-2.36 | 1.25 | 0.93-1.67 |
| **Female sex** | 1.18 | 1.07-1.30 | 1.20 | 1.08-1.33 |
| **White race** | 1.16 | 1.04-1.31 | 1.14 | 1.01-1.28 |
| **Diabetes mellitus** | 1.12 | 1.00-1.24 | 1.17 | 1.04-1.31 |
| **Age (per 10 years)** | 1.09 | 1.06-1.12 | 1.09 | 1.05-1.12 |
| **Cardiac Unit admission** | 1.07 | 0.88-1.28 | 1.26 | 1.02-1.55 |
| **Admission creatinine Cr >1-≤2 vs ≤ 1** | 0.94 | 0.85-1.05 | 0.85 | 0.76-0.96 |
| **Hypertension** | 0.91 | 0.82-1.00 | 0.87 | 0.78-0.97 |
| **Liver disease** | 0.84 | 0.70-1.01 | 0.90 | 0.73-1.10 |
| **Admission creatinine Cr >2-≤3 vs ≤ 1** | 0.76 | 0.61-0.95 | 0.71 | 0.55-0.91 |
| **Chronic kidney disease** | 0.70 | 0.59-0.82 | 0.80 | 0.66-0.96 |
| **Admission creatinine Cr >4-≤5 vs ≤ 1** | 0.56 | 0.31-1.03 | 0.57 | 0.31-1.07 |
| **Admission creatinine Cr >3-≤4 vs ≤ 1** | 0.37 | 0.22-0.62 | 0.36 | 0.21-0.62 |
| **Admission creatinine Cr >5 vs ≤ 1** | 0.24 | 0.11-0.53 | 0.33 | 0.14-0.75 |

The reference groups for ICU type, admission type, and admission serum creatinine were medical unit, ‘Other’ category admission type, and admission serum creatinine ≤ 1 mg/dL, respectively. Surgical ICUs are post-cardiac surgical, surgical, and trauma; medical ICUs refer to medical and cardiac. Adjusted odds ratios were calculated from a model including age, sex, race, ICU type, admission type, mechanical ventilation, comorbidities (hypertension, heart failure, CKD, diabetes and liver disease), and admission creatinine category.
