# Supplementary material for: Patterns of diuretic use in the intensive care unit
Source: PLoS One. 2019 May 31;14(5):e0217911. doi: 10.1371/journal.pone.0217911 (PMC6544280; doi:10.1371/journal.pone.0217911)
Supplement: S3 Table — The reference groups for ICU type, admission type, and admission serum creatinine were medical unit, ‘Other’ category admission type, and admission serum creatinine ≤ 1 mg/dL, respectively. Adjusted odds ratios were calculated from a model including age, sex, race, ICU type, admission type, mechanical ventilation, comorbidities (hypertension, heart failure, CKD, diabetes and liver disease), and admission creatinine category. (DOCX) [file pone.0217911.s004.docx]

**S3 Table.** Odds ratios for loop + thiazide use

| **Column1** | **OR for Loop +Thiazide** | **95% CI** | **Adjusted OR** | **95% CI** |
| --- | --- | --- | --- | --- |
| **Admission creatinine Cr >4-≤5 vs ≤ 1** | 9.04 | 6.76-12.08 | 6.09 | 4.41-8.41 |
| **Admission creatinine Cr >3-≤4 vs ≤ 1** | 7.75 | 6.19-9.70 | 4.87 | 3.76-6.29 |
| **Admission creatinine Cr >2-≤3 vs ≤ 1** | 5.65 | 4.77-6.69 | 3.28 | 2.69-4.01 |
| **Heart failure** | 4.66 | 4.17-5.20 | 2.60 | 2.29-2.95 |
| **Admission creatinine Cr >5 vs ≤ 1** | 3.89 | 2.73-5.56 | 3.21 | 2.19-4.71 |
| **Chronic kidney disease** | 3.82 | 3.41-4.28 | 1.46 | 1.26-1.68 |
| **Admission creatinine Cr >1-≤2 vs ≤ 1** | 2.60 | 2.28-2.96 | 1.84 | 1.59-2.12 |
| **Cardiac Unit admission** | 2.39 | 2.09-2.75 | 1.79 | 1.52-2.11 |
| **Mechanical Ventilation** | 2.36 | 2.10-2.64 | 2.73 | 2.40-3.10 |
| **Diabetes mellitus** | 2.15 | 1.93-2.40 | 1.32 | 1.18-1.49 |
| **Hypertension** | 2.14 | 1.90-2.41 | 1.41 | 1.24-1.61 |
| **Cardiovascular admission type** | 1.96 | 1.61-2.39 | 1.37 | 1.09-1.71 |
| **Respiratory admission type** | 1.62 | 1.27-2.07 | 1.13 | 0.87-1.46 |
| **Infectious admission type** | 1.60 | 1.25-2.05 | 1.04 | 0.80-1.35 |
| **Post-Cardiac Surgical Unit admission** | 1.41 | 1.21-1.64 | 1.11 | 0.92-1.35 |
| **Female sex** | 1.29 | 1.16-1.43 | 1.52 | 1.36-1.70 |
| **Age (per 10 years)** | 1.23 | 1.20-1.27 | 1.02 | 1.00-1.06 |
| **White race** | 0.93 | 0.82-1.04 | 0.98 | 0.86-1.11 |
| **Injury/Poisoning admission type** | 0.89 | 0.70-1.14 | 1.22 | 0.94-1.58 |
| **Liver disease** | 0.89 | 0.73-1.09 | 1.27 | 1.02-1.57 |
| **Gastrointestinal admission type** | 0.81 | 0.62-1.07 | 0.89 | 0.66-1.18 |
| **Surgical Unit admission** | 0.72 | 0.59-0.86 | 0.92 | 0.76-1.13 |
| **Neoplastic admission type** | 0.68 | 0.49-0.94 | 1.11 | 0.79-1.55 |
| **Trauma Unit admission** | 0.57 | 0.45-0.71 | 0.86 | 0.67-1.10 |

The reference groups for ICU type, admission type, and admission serum creatinine were medical unit, ‘Other’ category admission type, and admission serum creatinine ≤ 1 mg/dL, respectively. Adjusted odds ratios were calculated from a model including age, sex, race, ICU type, admission type, mechanical ventilation, comorbidities (hypertension, heart failure, CKD, diabetes and liver disease), and admission creatinine category.
